# Supplementary figures and images for: Reversible host cell surface remodelling limits immune recognition and maximizes survival of Plasmodium falciparum gametocytes
Source: PLoS Pathog. 2025 May 12;21(5):e1013110. doi: 10.1371/journal.ppat.1013110 (PMC12091884; doi:10.1371/journal.ppat.1013110)

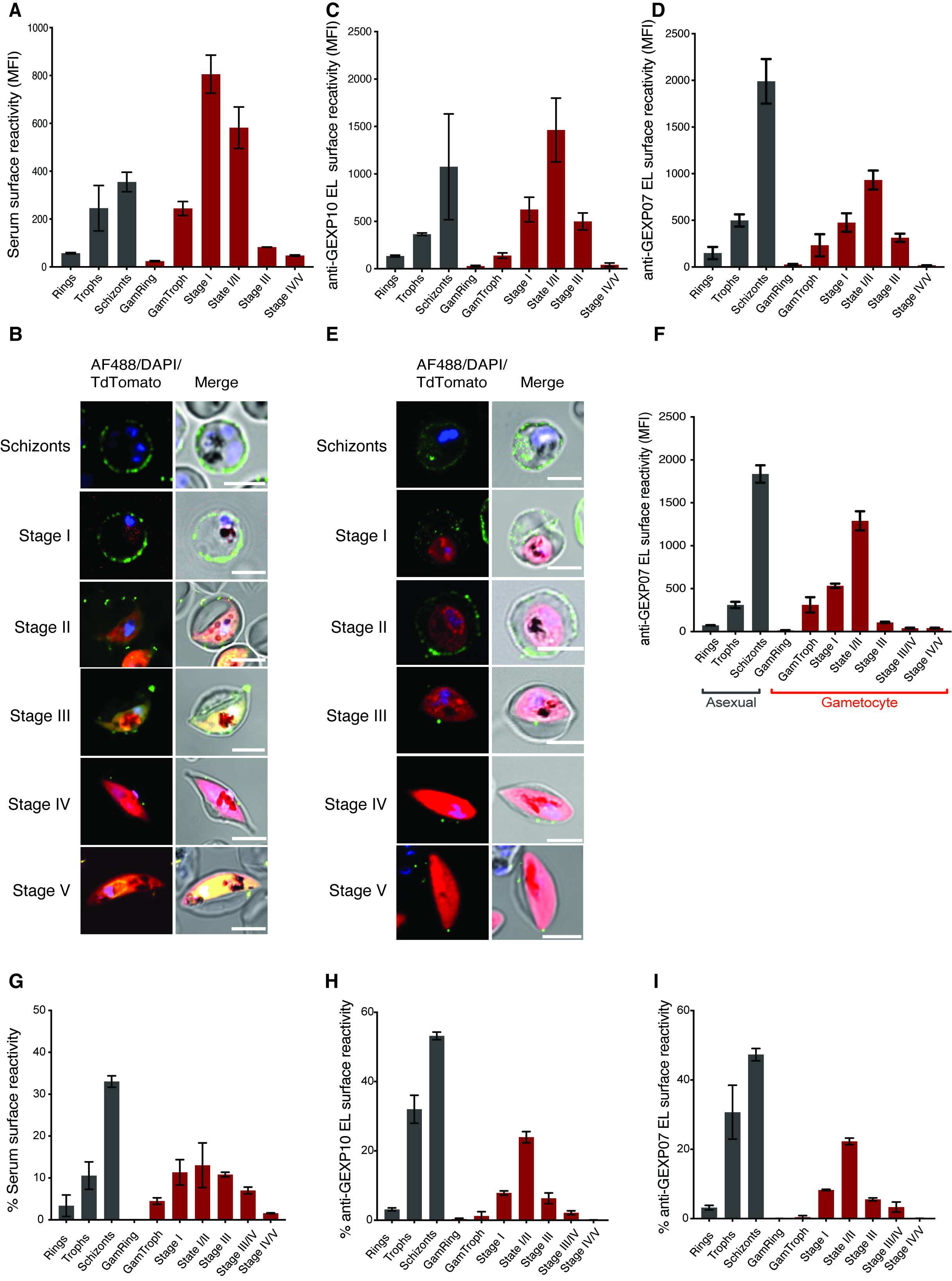

Supplement: S1 Fig — Detection of human serum reactivity in NF54 by flow cytometry (A) and live microscopy (B). C,D. GEXP10 EL (C) and GEXP07 EL (D) antibody surface reactivity in NF54. E,F. Reactivity of GEXP07 EL antibodies against live Pf2004 cells by live microscopy (E) and flow cytometry (F). G,H,I. Serum (G), GEXP10 EL (H) and GEXP07 EL (I) antibody reactivity in Pf2004 across asexual and gametocyte development by flow cytometry. Same data as in Figs 1B, 1D and S1F, respectively, but plotted as % positive cells. A-C, F-I represent the mean from three biological replicates. (TIF) [file ppat.1013110.s001.tif]

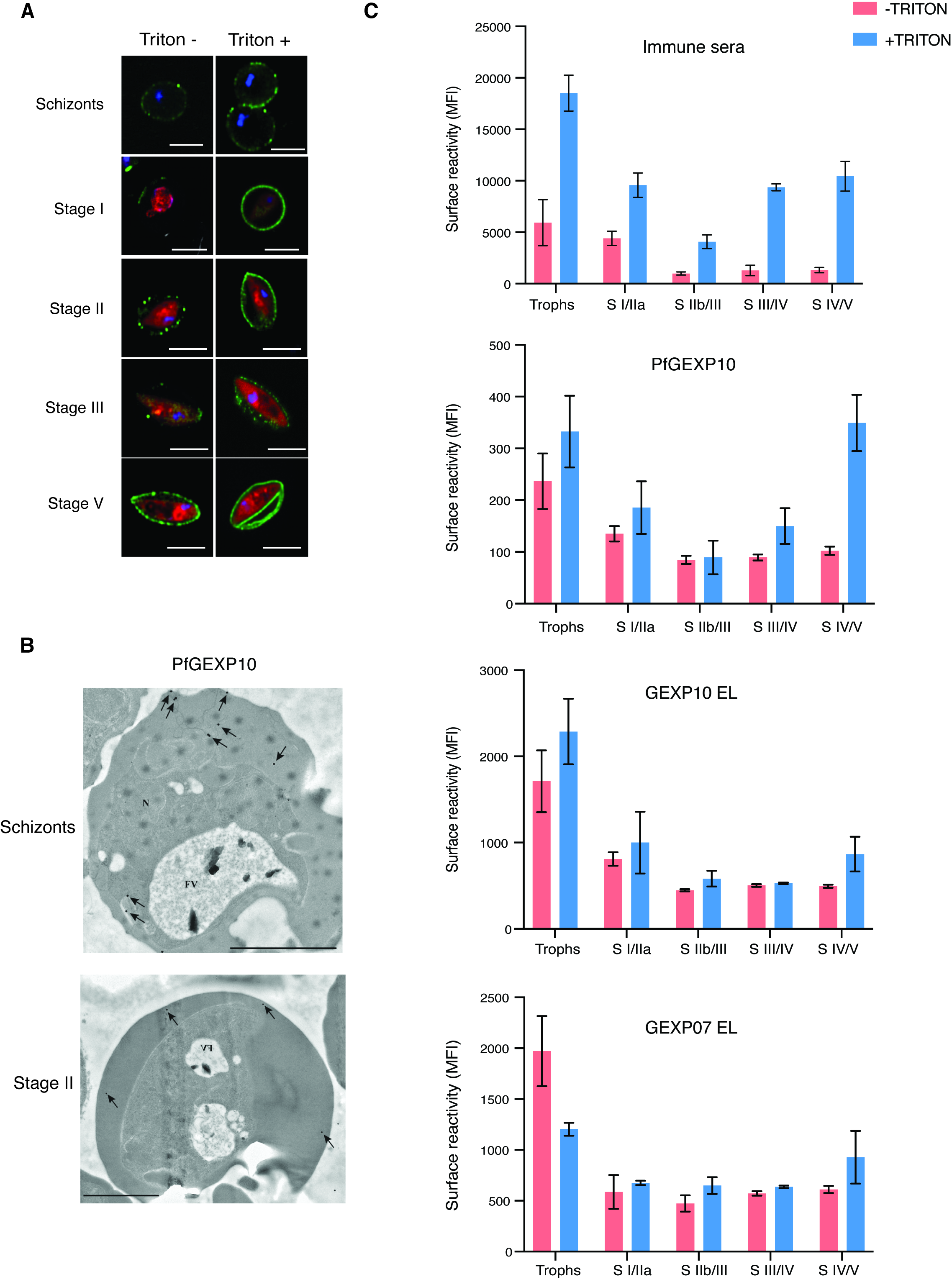

Supplement: S2 Fig — A. IFA data. GEXP10 EL surface labelling dynamics in fixed cells, internal labelling dynamics in fixed and permeabilized cells. B. Immuno EM with representative images of asexual schizonts and stage II gametocytes. A fraction of GEXP10 protein (based on GEXP10 antibody) is present on the surface of asexual and gametocyte iRBCs. C. Flow cytometry quantification of +/-Triton permeabilised cells. Asexual (24–36hpi), GI-Iia (D4), GIIb-III (D6), GIII-IV (D8), GIV-V (D11). Panels show from top to bottom immune serum, GEXP10, GEXP10 EL and GEXP07 EL reactivity. Blue: DAPI, green: IgG, red: Tdtomato. C represents the mean from three biological replicates. (TIF) [file ppat.1013110.s002.tif]

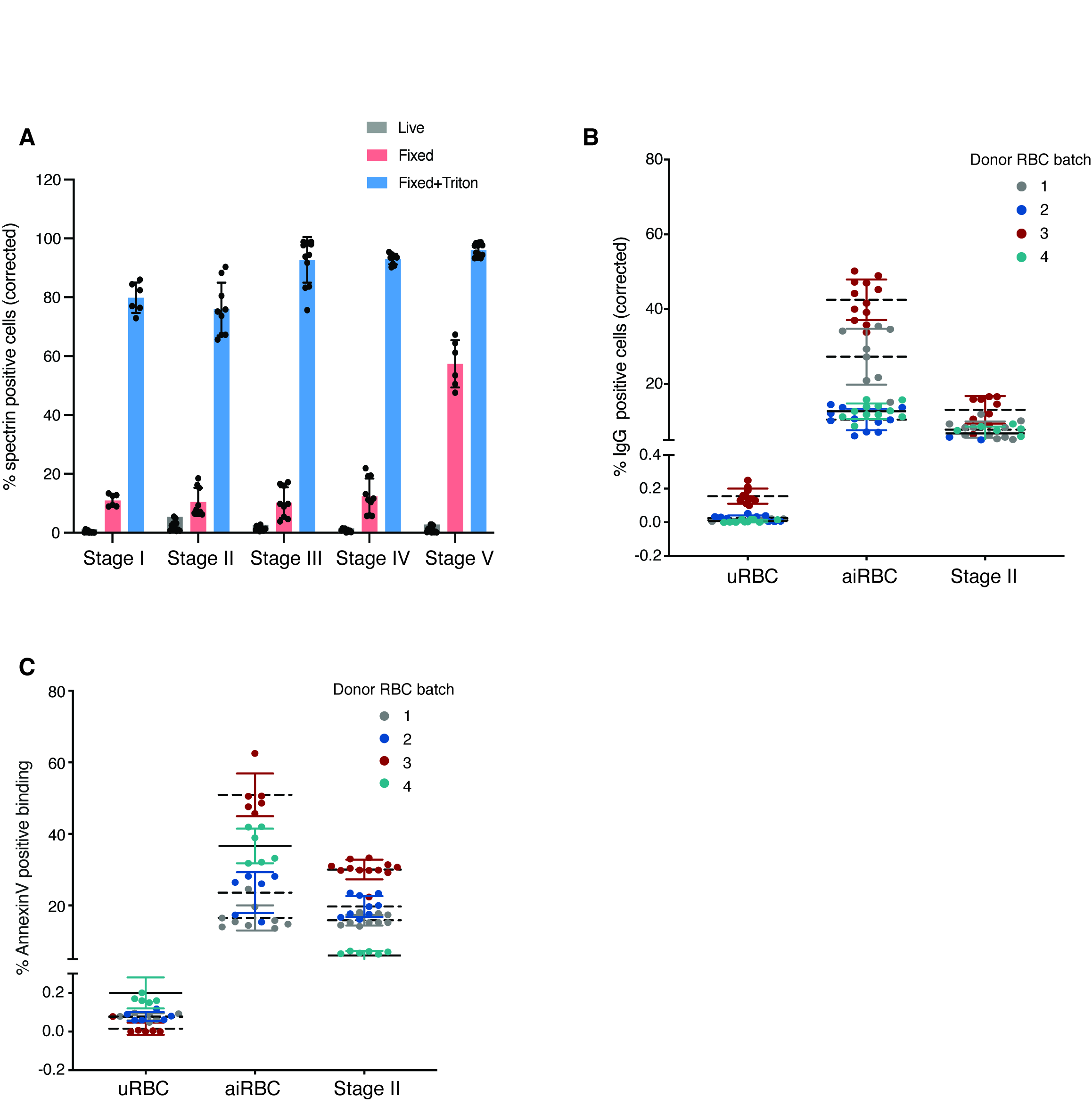

Supplement: S3 Fig — A. Dynamics of internal anti-spectrin antibody labelling in live, fixed only, and fixed and Triton permeabilised cells across gametocyte development. B,C. Variation of serum (B) and annexin V (C) labelling across experiments and using different donor RBC batches (1–4) for parasite culture. A represents the mean from two (stage I) and three (stage II-V) biological replicates. (TIF) [file ppat.1013110.s003.tif]

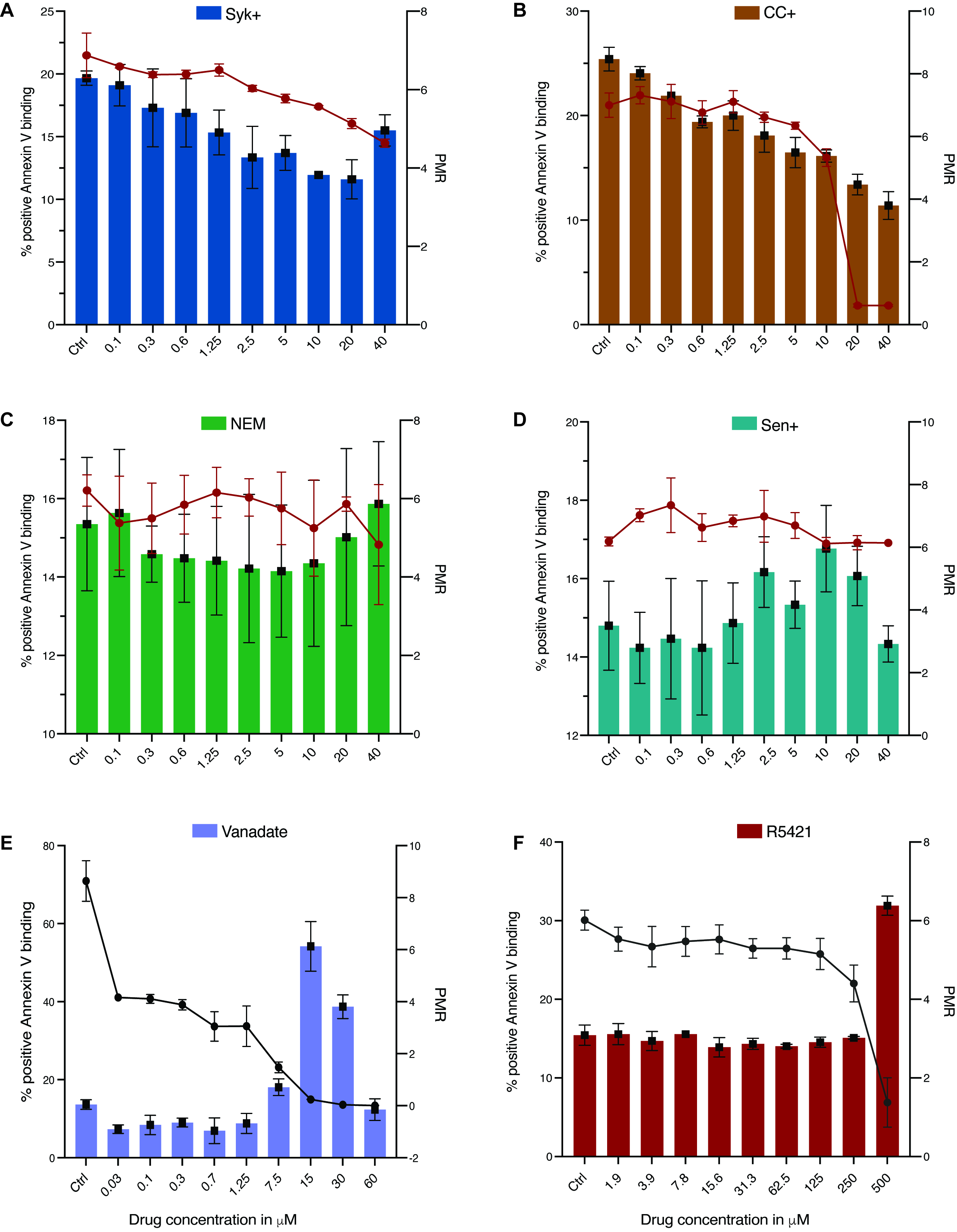

Supplement: S4 Fig — A-F. Testing various inhibitors to block PS exposure or internalization. (A) Syk inhibitor, (B) PKC alpha inhibitor (PKCa) – chelerythrine chloride, (C) Flippase inhibitor - NEM, (D) Senicapoc - Gardos channel inhibitor, (E) Vanadate - ATP inhibitor, (F) Scramblase inhibitor R5421/ oxalic acid dehydrate/ ethanoininic acid. A-F represent the mean from three biological replicates. (TIF) [file ppat.1013110.s004.tif]

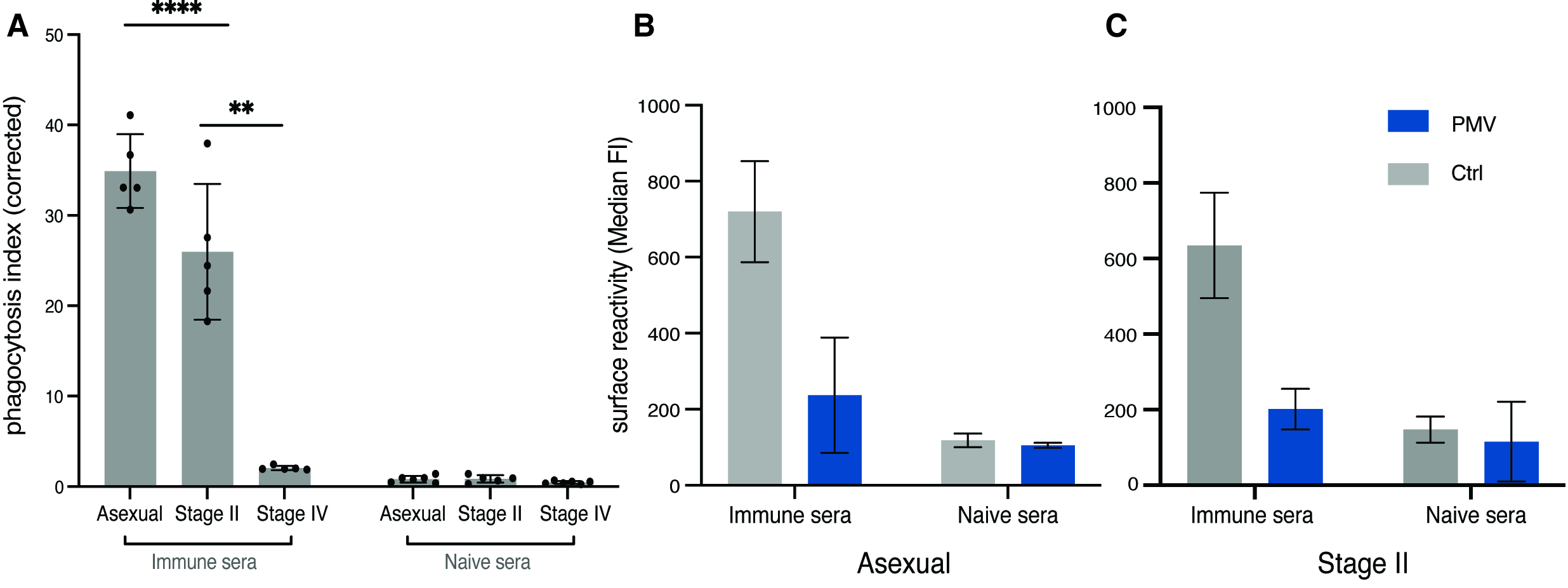

Supplement: S5 Fig — A. Control experiment using asexual, immature and mature gametocyte stages. Opsonisation with immune serum (left) and with US control serum (right). B,C. Effect of PMV inhibitor on opsonisation of asexual stages and immature gametocytes using immune serum vs US control serum. Same samples as in Fig 6A and 6B respectively. A-C represent the mean from three biological replicates. (TIF) [file ppat.1013110.s005.tif]
