# Supplementary material for: Reversible host cell surface remodelling limits immune recognition and maximizes survival of Plasmodium falciparum gametocytes
Source: PLoS Pathog. 2025 May 12;21(5):e1013110. doi: 10.1371/journal.ppat.1013110 (PMC12091884; doi:10.1371/journal.ppat.1013110)
Supplement: Attachment — Submitted filename: NgothoetalRebuttal.pdf [file ppat.1013110.s019.pdf]

Major comments:

Reviewer #2:

1. Although there is a significant difference in the annexin binding in the scramblase KO, it is conceptually challenging to understand why a scramblase localized to parasite internal membranes is playing a role in PS exposure in the host cell membrane. Although a fascinating observation, as it stands, the mechanistic insights into this phenotype is very limited. This might be challenging to address experimentally, however, a discussion of this unusual phenotype seems warranted.

Our data demonstrate that the activity of PfPL-SCR at the iRBC membrane requires the action of exported parasite proteins. While we cannot provide additional mechanistic insights at this point, we have added an example in the discussion for a scramblase acting in *trans*.

"Scramblases have been identified at various internal membranes, including ER, nuclear envelope and mitochondria and they can exert their function both in *cis* (i.e., in the same membrane) or in *trans*. For example, mammalian TMEM16K is localised at the ER membrane and regulates PS both in the ER and in organellar membranes of the endolysosomal system (Petkovic *et al*, 2020). Our data suggest that exported proteins facilitate the effect of PfPL-SCR1 at the iRBC membrane, as supported by the sensitivity of PS exposure to PMV inhibitor treatment."

2. The correlation between PS exposure and protein export in gametocytes is solely based on inhibitors with off-target side effects on other biological processes in the parasite, which might have downstream effects on PS exposure. To circumvent this, the authors could probe into export/PS correlation by direct interference with the translocon (using, for instance, conditional inhibition of PTEX components such as EXP2 or HSP101) to provide another line of evidence of this central point.

We apologise for the misleading statement regarding an off-target effect. The observed effect is not due to off-target activity, as the compound is basically a PEXEL motif peptide that outcompetes the natural substrate. No other protease has been shown to cleave PEXELs. Instead we propose a secondary effect, specifically a stress response due the blockage of the secretory pathway by the accumulation of uncleaved PMV substrate in the ER.

Additional phrases added to the discussion:

"It is therefore likely that a putative effect of PMV inhibitor on iRBC phagocytosis is masked by an unknown secondary effect that triggers phagocytosis independent of PEXEL-dependent parasite antigen exposure. The PMV inhibitor is essentially a PEXEL motif peptide that outcompetes the natural substrate. It is highly specific, and no other protease has been shown to cleave PEXEL peptides (Sleebs *et al*., 2014). Therefore, an off-target effect is unlikely. As blocking PEXEL cleavage accumulates substrate antigens in the ER and jams up the secretory pathway, it is most likely that the PMV inhibitor induced a stress response that altered the iRBC surface."

3. For quantitative localization/export data, the use of endogenously tagged marker proteins instead of the provided antibody-based IFAs/EM is advised.

We agree that tagged GEXP proteins would be very useful for localization studies. However, we are concerned that the reporter will alter localization of these proteins. Specifically, the two transmembrane proteins GEXP07 and GEXP10 were localised to the iRBC surface by Philippe Deterre and colleagues (Hermand *et al*., Sci Rep 2016) using peptide-specific antibodies. We have confirmed this localization with their antibodies and our own polyclonal GEXP10 antibody in a previous study (Dantzler *et al*., Sci Trans Med 2019) and in this study. In contrast, two groups have used a C-terminal GFP reporter to tag both proteins and could only detect them in RBC internal Maurer's clefts membranes (McHugh *et al*., mBio 2020 and Jennison *et al*., Cell Rep 2019). We assume that the reporter abrogates surface localisation of the protein and instead arrests it at the Maurer's clefts. We therefore conclude that tagging will not be helpful to investigate the localization of these proteins.

Reviewer #3:

1. The authors use CBPI, CBP2, GEXP7 and GEXP10 interchangeably, which is very confusing. In figure 1F, one set of columns is labeled as CBPI, another as GEXP10. If these are different proteins, the authors should explain this clearly and define the proteins more clearly. If they are not different proteins, it would

be very useful for the reader to stick with one. In some cases, the text will use one name of the protein and figure the other.

Apologies for the confusion. The proteins were originally annotated as GEXP07 and GEXP10 by Silvestrini *et al.* (Mol Cell Prot, 2010). They were then renamed into CBPI (GEXP10) and CBPII (GEXP07) by Hermand *et al.* (Sci Rep, 2016). We obtained their peptide antibodies against the extracellular loop and accordingly kept the CBP annotation, while we named our own ab as GEXP10. We agree that this is confusing and have renamed all antibodies as GEXP07 and GEXP10, in line with the annotation in Dantzler *et al.* (Sci Trans Med, 2019). They are now named as GEXP07 EL (EL = extracellular loop) and GEXP10 EL for the peptide antibodies by Hermand *et al.*, and GEXP10 for our own antibody. Text and figures have been corrected accordingly.

2. It is difficult to understand the difference between Annexin V positive and Annexin V negative (Figure 2B) without seeing a representative plot. The authors should include an example of their cytometry result so that the reader can understand how different positive and negative cells are on cytometry plot.

We thank the reviewer for this suggestion. We have now included the scatter plots for the annexin V flow cytometry data showing how we gated the Annexin V positive population in revised figure 2A.

3. It is unclear what the 'samples' in Figure 3A represent. Are these individual experiments? Does % positive refer to the percentage of positive cells within one samples? If so, please indicate how many cells were counted per sample.

We have added the following sentences to the legend of Figure 3A:

"Each point represents the mean of 3 biological replicates of the combined data from the serum (Figure S1G) and PS exposure (Figure 2A) time course experiments. For each experiment a minimum of 10,000 cells was acquired."

Reviewer #4: None

Minor comments:

Reviewer #2: (No Response)

Reviewer #3:

The results in Figure 2D would be clearer if the authors false-coloured either the PS 674 or the TdTom white. Currently, the magenta and red are difficult to differentiate.

Thanks for this suggestion. We have changed the coloring accordingly.

In figure 3B, the labeling of iv-vi is missing.

Done.

In the legend for Figure 3, 'PMV' is mentioned when 'PMV inhibitor' is likely meant.

Done.

Line 433: The statement that this provides a proof of concept is overstated without any transmission experiments. Only a small percentage of infected erythrocytes is affected, so it remains to be determined whether targeting PS exposure affects transmission significantly.

We already argue that the low proportion is an in vitro artefact. I think the statement is OK. Priscilla?

Line 536 1500rpm would be better described in x g.

Done.

Line 544 'data was' should be 'data were'

Done.

The NEM used for the experiment shown in Figure 5A may also affect protein transport. The authors should comment.

We did not pursue NEM because it had a major effect on gametocyte development, i.e. cells were arrested.

The difference between the control and the NEM sample in Figure 5C is very large. Is the significance of that really only *? The number of * is not defined in any figure legend, an explanation of the value of the different number of * should be added.

Thanks for pointing out this error. We have updated Figure 5C with the correct statistics and explained the asterisks in the legend.

"Statistics on B-C represent paired T test on inhibitor treated *vs* untreated (DMSO only) control. *p*-value <0.0001

****, *p*-value <0.001 ***, *p*-value ≤ 0.01 **, *p*-value ≤ 0.05 * and *p*-value > 0.05 ns".

Reviewer #4:

1. In Figure 1 the authors show that the majority of the parasite surface antigens remain internal and suggest that this may imply another function. Do the authors know whether these proteins are exported to the PVM or the host cell cytoplasm as they could then act in an immune evasion mechanism post red blood cell rupture?

We agree that the internal antigen pool likely has additional functions. For example, it has been demonstrated that GEXP07 and Stevor antigens have structural roles within the host cell.

Additional text has been added to the discussion:

The internal antigen pool may also serve additional structural roles within the host RBC, as has been demonstrated for GEXP07 (McHugh *et al*, 2020) and for STEVOR antigens (Naissant *et al*, 2016).

2. A key experiment is the use of a PMV inhibitor that is known to impact export of exported parasite proteins. However, the data seems to indicate that the inhibitor does not prevent recognition of gametocyte by immune sera. Does this suggest that there are potentially surface antigens that are not dependent on PMV for its export - this could be explored a bit more in the manuscript.

See response to reviewer #2.

3. Fig5 and line 266 - as far as I understand the inhibitor was added on day 3 and annexin staining was measured on day 6 and this showed no apparent reduction in staining as compared to the control. However in Figure 2 the authors show that annexin V staining is already near its peak in stage I gametocytes. This would suggest that the inhibitor might be already added to late. Can the authors clarify this?

Thanks for poiting this out. Parasites were treated on day 2 (and not on day 3 as originally stated) for 2 consecutive days. This has been updated in the text as below.

“To block PS surface flipping at stage II gametocytes parasite were treated with the inhibitors on day 2 post induction (early stage I gametocytes) for 2 consecutive days.”

4. In the discussion l 326 the authors suggest that inhibition of surface expression may present a potential therapeutic strategy as this would make gametocytes more accessible to immune recognition and therefore clearance. At the same time they state throughout the manuscript that surface expression of parasite antigens is important for immune recognition. While I think I understand what the authors try to indicate this is somewhat confusing.

We agree that this phrase is contradictory and removed it from the discussion.

5. There seems to be a discrepancy (line 400) between this work and the published work by Dantzler et al. in relation to the role of parasite surface antigen expression and phagocytosis - while I understand that experimental differences could account for this it would be beneficial for the reader if this is clearly described.

We assume this comment has been resolved with the additional text regarding the activity of PMV inhibitor.

6. The Marti lab as well as others have previously published work that indicates differences in the rigidity of the maturing gametocyte and splenic retention. based on the work here would it make sense that the initial surface expression of parasite antigens is key for targeting the bone marrow niche and that the retention is then driven by biomechanic differences. It would be of value of the authors consider addressing these two aspects in the discussion.

Thanks for this suggestion. We have added a phrase in the discussion:

“We propose a role in cytoadhesion that supports retention of the rigid immature gametocyte in the extravascular environment.”
