# Supplementary material for: Reversible host cell surface remodelling limits immune recognition and maximizes survival of Plasmodium falciparum gametocytes
Source: PLoS Pathog. 2025 May 12;21(5):e1013110. doi: 10.1371/journal.ppat.1013110 (PMC12091884; doi:10.1371/journal.ppat.1013110)
Supplement: Attachment — Submitted filename: 146674_1_rebuttal_3125186_sqptzw.docx [file ppat.1013110.s017.docx]

**Manuscript number:** RC-2024-02498

**Corresponding author(s):** Matthias, Marti

*[The “revision plan” should delineate the revisions that authors intend to carry out in response to the points raised by the referees. It also provides the authors with the opportunity to explain their view of the paper and of the referee reports.*

*The document is important for the editors of affiliate journals when they make a first decision on the transferred manuscript. It will also be useful to readers of the reprint and help them to obtain a balanced view of the paper.*

*If you wish to submit a full revision, please use our "*[*Full Revision*](https://reviewcommons.org/templates/full-revision.docx)*" template.* ***It is important to use the appropriate template to clearly inform the editors of your intentions.****]*

## 1. General Statements [optional]

*This section is optional. Insert here any general statements you wish to make about the goal of the study or about the reviews.*

We have recently reported reversible remodeling of the infected red blood cell surface with parasite antigens during *P. falciparum* gametocyte development, resulting in immune reactive immature stage and immunologically silent and transmission-competent mature stages. In this study we aimed to further investigate this process and its translational implications.

We are pleased to read the overall positive reviews, noting that “the results advance our understanding the interaction of the parasites with the host cell and point to a new form of antimalaria intervention.” The reviewers also raised some concerns that we have addressed with a series of experiments that are already included in this preliminary revision, and with additional experiments that are ongoing. Altogether we believe the suggested changes (including the ongoing experiments) will greatly improve clarity and significance of this manuscript.

## 2. Description of the planned revisions

*Insert here a point-by-point reply that explains what revisions, additional experimentations and analyses are planned to address the points raised by the referees.*

***Ongoing experiments***

**1. Both reviewers comment on the localization of the parasite scramblase at the parasite host interface vs its function in PS flipping on the infected RBC surface.**

***Rev 1:*** *The authors should check the localization of the scramblase at the RBC membrane to support their conclusion that "the parasite scramblase is required for efficient PS flipping on the iRBC surface in asexual and early gametocyte stages" (l.200).*

***Rev2:*** *Although the data in Figure 4 that PfPL-SCR1 has a function in the presentation of phosphatidylserine on the surface of the infected erythrocyte are somewhat compelling, it is very unclear how the protein may perform this function. The authors should speculate how a protein present within the parasite affects the distribution of phospholipids on the surface of the erythrocyte, as it is not immediately obvious. Is it possible that the parasites lacking PfPL-SCR1 have a growth delay that does not affect the production of the viable Stage V gametocytes? Also, the percentage of positive cells is much lower than the percentage of positive cells in Figure 2B. The authors may want to comment.* 

We are currently imaging PfPL-SCR1 in GFP parasites to clarify its localization either in the parasite PM or in the PVM. Data will be presented in the full revision of the MS. In addition, we have added text in the discussion regarding the putative function of PfPL-SCR1.

**2. It was noted that the effect of gametocyte infected RBC phagocytosis in the PfPL-SCR1 KO line is missing.**

***Rev1****: Figure 6: The authors should monitor whether iRBC uptake by THP-1 cells is affected in the PfPL-SCR1 KO line at the gametocyte stage, and not only at the asexual stage.*

We are currently quantifying the efficiency of gametocyte phagocytosis in PfPL-SCR1 KO parasites vs WT for comparison with asexual stages. The data will be presented in the full revision of the MS.

**3. It is noted that gametocyte stage annotation is incorrect.**

***Rev1****: In different figures (figures 1C, 1E, 2C, 2D, 3H): Stage V do not present the typical banana shape and look like immature stages. Are the authors sure about the maturity of the tested parasites? If yes, the figures should show parasites that are representative of the population used for the experiment.*

The reviewer is correct that images are not always representative. We will replace these images and show representative images of Stage V with the NF54 reporter line. Please note that our Pf2004 reporter line has a gametocyte maturation defect, where Stage V gametocytes do not look like the typical banana-shaped cells as in NF54. Data will be presented in the full revision of the MS.

## 3. Description of the revisions that have already been incorporated in the transferred manuscript

*Please insert a point-by-point reply describing the revisions that were already carried out and included in the transferred manuscript. If no revisions have been carried out yet, please leave this section empty.*

***Reanalysis (already carried out)***

***Rev 1:*** *Figure 1: A control is missing to ensure that the infected RBC are not permeable to antibodies. The authors should use spectrin staining as a negative control for surface antigen exposure (the antibody epitope is in the RBC cytoplasm). This would exclude false positive signal and staining of permeable cells in the absence of triton detergent.*

We have performed the suggested control experiments with spectrin antibodies and demonstrate that live iRBCs have intact membranes, i.e. antibodies are not leaking into the cell and hence there is no internal labeling. These new data are included in new **Figure S3A**.

***Rev 1****: Only a subpopulation of RBC infected with immature gametocytes is annexin-positive and there is a high variability between experiments. In Figures 2A and 2B, at its peak of exposure, only 30% of the cells exhibit PS surface exposure; in Figures 3J and 4C, the percentage of Annexin V-positive cells appears even less significant, with 15% in the control and 6% in the WT conditions, respectively. Since the normal ageing process of RBC results in PS exposure, we cannot exclude that the variability is related to the age of the RBC used for the experiments. To exclude this possibility, experiments should be performed with RBC of the same age, following a classical protocol of RBC separation according to their age on a density gradient.*

***Rev1:*** *The authors state that there is a difference in PS exposure in Stage II and Stage V gametocytes, however this statement is not supported by the data when we compare different figures: in Figure 5B percentage of Annexin V-positive cells in the untreated condition is nearly 20%, which is more than observed for Stage II in Figures 3J and 4C.*

***Rev 1****: To support that "surface antigen expression ... is on in immature and off in mature stages" (l 34-36), the authors should show the proportion of cell showing surface antigen expression in immature stages.*

These three comments by reviewer 1 address an important issue that we have observed throughout the experiments. Specifically, we have noted that only a subpopulation of 15-30% of iRBCs are Annexin V positive at peak time, as has previously been reported by Fraser *et al* (PLoS Path, 2021). As suggested by the reviewer, we have now included an uninfected RBC control in the analysis to exclude possible contribution of RBC age to the observed PS levels (due to eryptosis). These data demonstrate that the uninfected RBCs controls only expose background PS levels on the surface. Data are included as new **Figure** **S3B**. The same figure also reveals that differences across RBC donor batches are the major determinant causing the observed variation in iRBC PS levels (both asexual and gametocyte stages).

Importantly, the same applies to iRBC surface staining with human serum or specific antibodies: less than 40% of cells is positive (in contrast to greater 90% with the anti-RBC control antibodies) even at peak time (i.e., Stage II gametocytes) as we have previously observed (Dantzler et al, STM 2019). This is also confirmed by various observations with PfEMP1 staining on the iRBC surface of asexual iRBCs (e.g. Williams *et al*, Cytometry 2003). As with PS, there is also major variation in iRBC staining across RBC donor batches (**Figure S3C**).

Altogether these additional experiments identify RBC donor batches as the major determinant for the observed variation of iRBCs with PS exposure and IgG reactivity across experiments. In addition, technical limitations in the detection of fluorescence signal on the surface of live cells and the relatively low abundance of parasite antigens on the iRBC surface contribute to low MFI and % positive rates in our experiments. We hypothesize that PS and antigen surface exposure are essential for the survival of immature gametocytes. Therefore, we assume that the variable and low percentage of iRBCs with PS exposure and IgG reactivity is an artifact of parasite *in vitro* culture. A paragraph both in the results and discussion have been added to highlight these observations.

In addition to the above experiments, we have also plotted Annexin V staining as % positive relative to control throughout the figures to normalize for variation across blood donor batches and experiments. We have also included a series of alternative graphs in **Figure S1** that show the % positive IgG for serum and antibody instead of MFI.

***Rev1:*** *The data shown in Figure 1E does not support the conclusion that "reversible surface exposure of parasite antigens peak(ing) at stage II gametocytes": Figure 1E shows that surface antigen recognition is minimum at Stage II in comparison with the other stages without permeabilization.*

We have rephrased this sentence as suggested. The point of this figure is that antigen production is continuous, but iRBC surface exposure is restricted to stage I-II.

***Rev1****: Figure 5: inhibitor concentrations should be tested on gametocyte viability, since these concentrations likely differ in asexual stages and in gametocytes.*

This is an interesting suggestion. Unfortunately, we do not have the tools available yet to do this experiment. While viability markers for asexual parasites are well established, there is currently no gold standard for gametocytes.

***Rev1:*** *Figure 6D, 6E: What means uncorrected and corrected phagocytosis index?*

We agree that this has been unclear and changed the y-axis to opsonic phagocytosis index and non-opsonic phagocytosis.

***Rev2****: The authors do a very good job investigating the mechanisms of the change of phosphatidylserine presentation on the surface of the host cell, but it seems like a trick missed that the mechanisms of the change in the level of antigens is not investigated. At least some mention of a potential mechanism how surface proteins are lost from the surface of the erythrocyte when the parasite reaches stage V would be informative (and an experiment testing protease inhibitors would be even better, but not required).*

We thank the reviewer for this suggestion. We have now included a paragraph regarding the possible mechanisms of reversible antigen exposure in the discussion (e.g., protease cleavage, membrane remodeling, vesicle release).

***Rev2:*** *Recent papers (and preprint) from the Goldberg, Ishino, van Ooij, Beck and Muralidharan labs have shown that Plasmepsin V has a function beyond processing exported proteins. Hence, the observed effects of the PM V inhibitor on phosphatidylserine surface exposure may be the results of the lack of processing of a non-exported protein (Figures 3J and 3K).*

We thank the reviewer for this suggestion and have added some text and the suggested references in the discussion.

***Rev2****: The authors present data based on immune-EM images, but no representative image is shown. For the reader to have a sense what the data compiled in the graph in Figure 1F look like, it would be useful to include representative images.*

The EM data are in supplementary data S2B and referenced in the main text. We can provide more representative images if requested by this reviewer or the editor.

***Rev2:*** *In Figure 2C, Stage I panel, the parasite itself is stained. This may either indicate that there is a connection from the PV to the outside of the erythrocyte or that the cell shown was permeabilized. A comment on this would be useful.*

This is well spotted by reviewer 2. We have replaced this image with a proper representative image in Figure 2C.

**Rev1,2:** All suggested minor text changes were incorporated.

## 4. Description of analyses that authors prefer not to carry out

*Please include a point-by-point response explaining why some of the requested data or additional analyses might not be necessary or cannot be provided within the scope of a revision. This can be due to time or resource limitations or in case of disagreement about the necessity of such additional data given the scope of the study. Please leave empty if not applicable.*

*N/A*
